# Supplementary material for: Nanoscale chemical reaction exploration with a quantum magnifying glass
Source: Nat Commun. 2024 Jun 22;15:5320. doi: 10.1038/s41467-024-49594-2 (PMC11193806; doi:10.1038/s41467-024-49594-2)
Supplement: Supplementary file 1 — Supplementary Information [file 41467_2024_49594_MOESM1_ESM.pdf]

SUPPORTING INFORMATION

Nanoscale chemical reaction exploration with a quantum magnifying glass

Katja-Sophia Csizi[a, ‡], Miguel Steiner[a, b, ‡], Markus Reiher[a, b, *]

[‡] Authors contributed equally

[*] Corresponding author; e-mail: mreiher@ethz.ch

[a] ETH Zurich, Department of Chemistry and Applied Biosciences, Vladimir-Prelog-Weg 2, 8093 Zurich, Switzerland

[b] ETH Zurich, NCCR Catalysis, Vladimir-Prelog-Weg 2, 8093 Zurich, Switzerland

1 Details on QM/MM structure and transition state optimization routines

Due to the many degrees of freedom to be optimized in large systems, QM/MM structure optimizations with QM/SFAM were carried out in a micro-iterative fashion. First, all Cartesian coordinates of the QM region and those of the environment atoms bound to atoms in the QM region were frozen (micro-iteration step with N_1 steps). All remaining MM degrees of freedom were relaxed until convergence (or until a maximum number of iterations is reached). Second, the complete system was relaxed by applying the full analytical QM/SFAM gradients (macro-iteration step with N_2 steps). The procedures were iterated until convergence was reached. The optimization of transition states involves the costly calculation of the Hessian matrix $\mathbf{H}$. In the QM/MM formalism, the full Hessian matrix reads

$$\mathbf{H}_{\mathrm{QM/MM}} = \begin{pmatrix} \mathbf{H}^{\mathcal{Q}} & \mathbf{H}^{\mathcal{E}-\mathcal{Q}} \\ \mathbf{H}^{\mathcal{Q}-\mathcal{E}} & \mathbf{H}^{\mathcal{E}} \end{pmatrix}. \tag{1}$$

Here, the diagonal blocks $\mathbf{H}^{\mathcal{Q}}$ and $\mathbf{H}^{\mathcal{E}}$ denote the second derivatives with respect to QM and MM atom coordinates, respectively, and the off-diagonal terms correspond to the mixed second derivatives with respect to one QM and one MM atom coordinate. In practice, the calculation (and storage) of the full Hessian matrix is computationally demanding, at least when electrostatic embedding is applied, because many coupled-perturbed self-consistent field equations need to be solved (semi-numerically) for the displacement of each MM atom. We therefore resorted to the partial Hessian approach, in which the MM degrees of freedom are neglected and only a sub-block of the full Hessian matrix is diagonalized to obtain vibrational frequencies[1]. Provided that the eigenvectors for the vibrational modes of interest are localized on the atom block for which diagonalization is carried out, good agreement with experiment can be achieved[2]. To exploit the partial Hessian ansatz in a transition state optimization routine, we implemented a QM/MM transition state optimizer inspired by the micro-iterative QM/MM structure optimizer, as shown in Supplementary Figure 1.

First, the transition state guess molecular structure is divided into the QM region $\mathcal{Q}$ and the environment region $\mathcal{E}$. We dynamically change the embedding scheme between the separate optimization routines. That is, we apply mechanical embedding when $\mathcal{E}$ is optimized, and electrostatic embedding when $\mathcal{Q}$ is optimized. During MM optimization, the QM degrees of freedom at the boundary are fixed. Convergence is considered reached once the QM optimization converged twice in a row, i.e., before and after an MM optimization to ensure self-consistency.

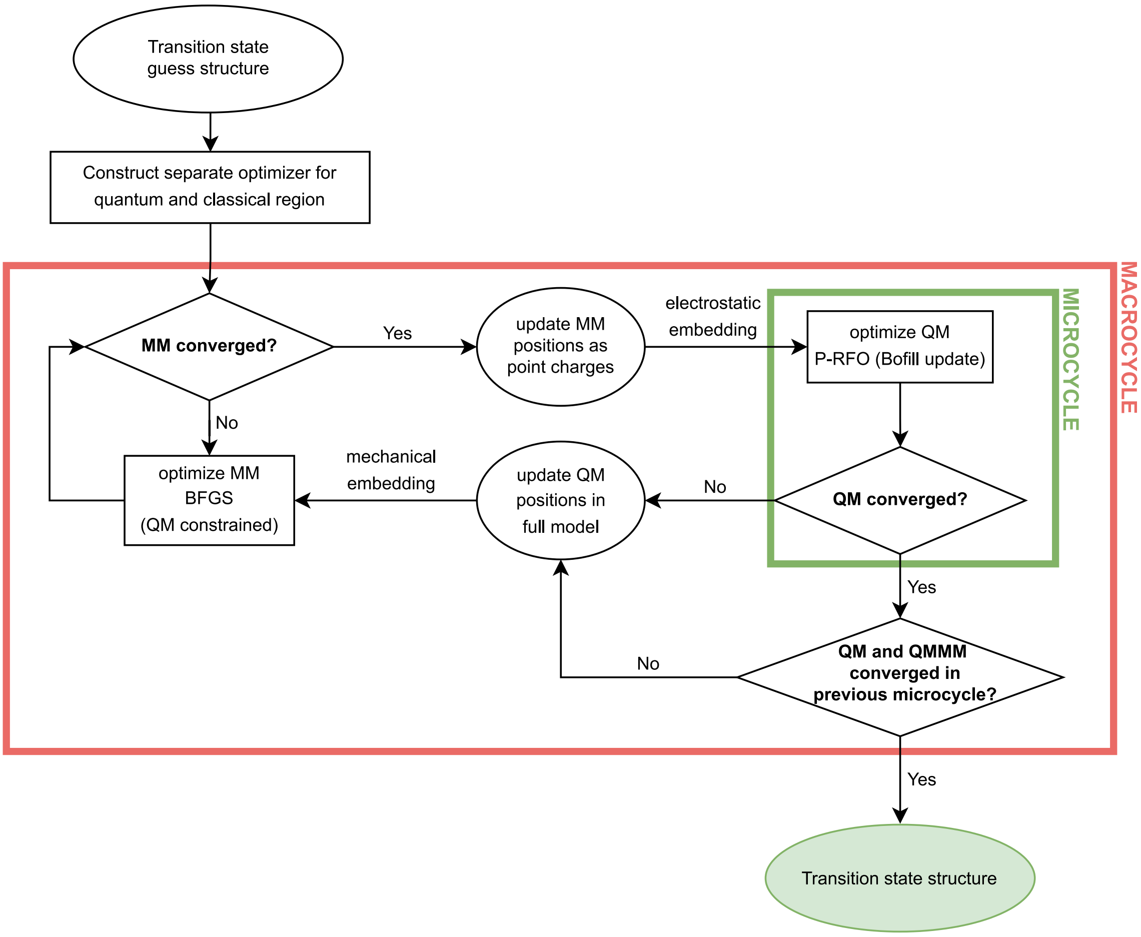

Supplementary Figure 1: Schematic representation of the quantum mechanical/molecular mechanical (QM/MM) transition state optimization routine developed and implemented in SCINE UTILITIES for this work. The algorithm exploits a partial Hessian scheme to efficiently optimize the atoms in the QM region to yield a transition state while optimizing the atoms in the MM region to an energy minimum without the need of Hessian calculations in the MM region. This is achieved by a partitioned rational-function optimizer (P-RFO)[3] with a Bofill update[4] for the QM region, commonly referred to as eigenvector following[5], and a Broyden–Fletcher–Goldfarb–Shanno (BFGS)[6–9] optimizer for the MM region. In general, any optimizer in the SCINE software framework, such as the dimer optimizer and a limited memory implementation (L-BFGS), can be applied as well. The changed positions of the QM optimization are applied to the full model by direct position changes, while the changed positions of the MM-atom optimization are applied to the subsequent QM optimization by changing the point charges to match the new MM region.

2 Assessment of SFAM parameters

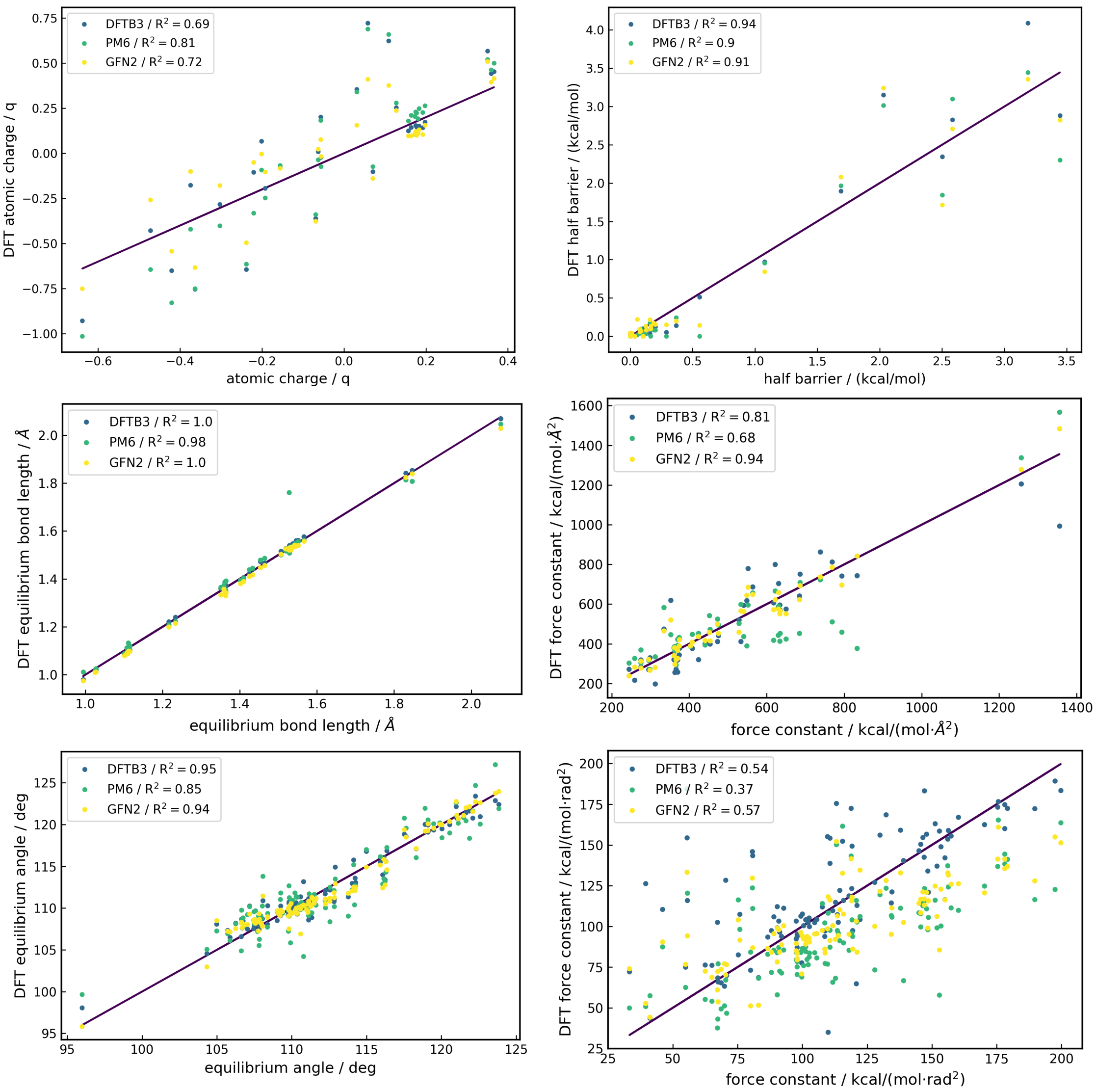

Supplementary Figure 2: Correlation plots of all SFAM parameters obtained for different atom types in the SFAM model of insulin chain A derived from different reference methods. For details on the functional form of SFAM see Ref. 10.

To assess the dependence of SFAM parameters on the given reference method, the parameter sets obtained from semiempirical reference data can be compared to the DFT-derived SFAM model. We find excellent agreement in the structural parameters derived from the different models, (equilibrium bond lengths and angles) indicating that sufficiently reliable reference structures are obtained with all semi-empirical methods. With regard to the obtained force constants and half barrier heights, we find excellent agreement between DFTB3 and GFN2-xTB and the PBE-D3 reference for the covalent bond terms ($R^2 \geq 0.81$) and, among all semiempirical methods,

for the dihedral half barrier heights (R^2 ≥0.91). For the angle force constants, we find the largest spread among the models, where DFTB3 most closely matches the PBE-D3 reference (R^2=0.54). With regard to the atomic charges, we converted the different charge models of the respective QM models to Charge Model 5 (CM5) charges to ensure consistency, but defer a detailed investigation of possible disadvantages to future work. Overall, our findings of parameter accuracy point towards DFTB3 as the most accurate semiempirical model for the given system. However, we stress that force field parameters must be compared with caution, because any system-focused force field is deliberately constructed to reproduce the potential energy surface of the reference method accurately, at least close to the minimum energy structure. To gradually improve the initial parameters of a given SFAM force field, it is possible to train an ML model on additionally generated reference data during sampling of many different states (for instance by means of an MD simulation)[10].

3 Resources requirements for SFAM parametrization

For reference data generation of DFT quality, the mean serialized central processing unit (CPU) computing time amounted to 5.7 h, and the total CPU time to 3856.2 h. For reference data generation of GFN2-xTB quality, the mean CPU time amounted to 0.50 min, and the total CPU time to 9.6 h. For reference data generation of DFTB3 quality, the mean CPU time amounted to 0.18 min, and the total CPU time to 3.6 h.

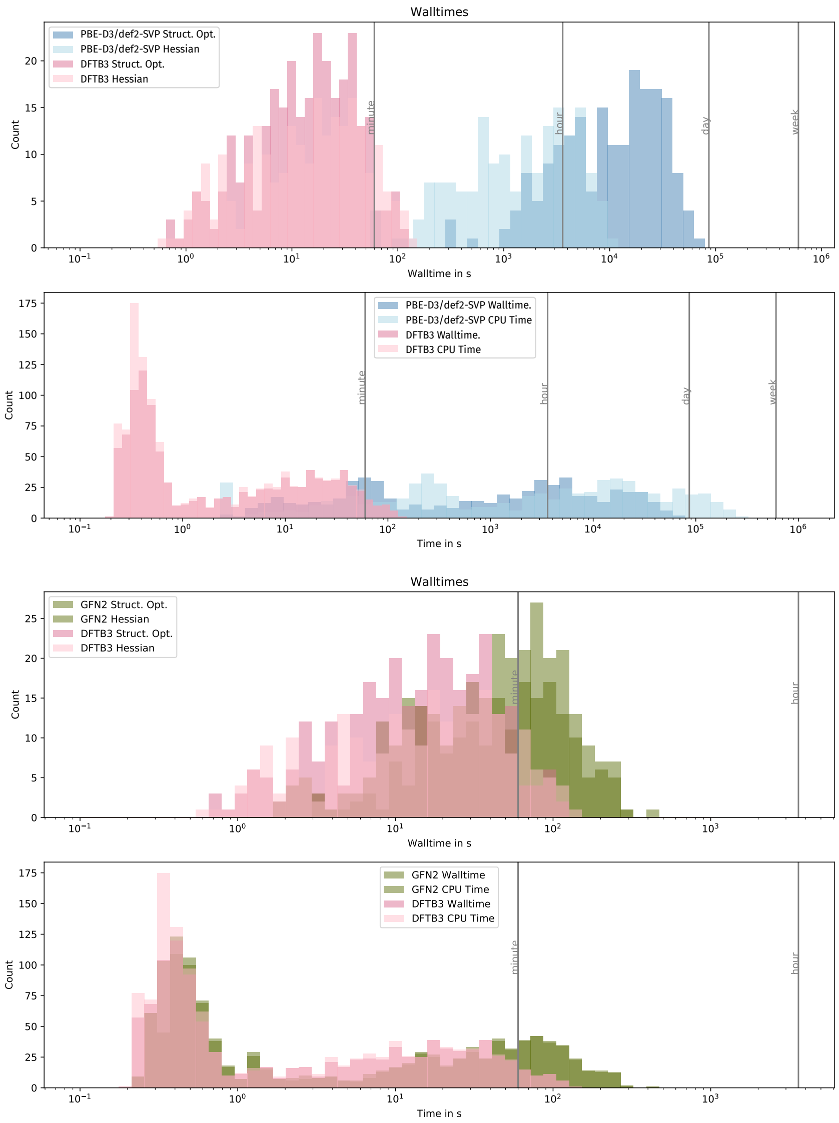

Supplementary Figure 3: Distribution of runtimes for the parametrization of insulin with DFTB3 (pink), PBE-D3/def2-SVP (blue), and GFN2-xTB (green).

4 Additional data on esterification in insulin

4.1 One-step reaction mechanism

In the core model, we defined a set of rules to steer the reaction and truncate the search space. All atoms in carboxylate- and hydroxyl-groups were selected to be reactive. In Scine Chemoton, this reactivity can be encoded by defining reactive atom types by their coordination environment. We defined the following atoms to

be reactive:

1. all hydrogen atoms bound to an oxygen atom.
2. all oxygen atoms in hydroxyl groups (that are all oxygen atoms bound to one carbon atom and one hydrogen atom).
3. all oxygen atoms in a carboxylic acid group (that are all oxygen atoms bound to one carbon which itself is bound to three other atoms, two of them being oxygen atoms and one being another carbon atom).
4. all carbon atoms in carboxylic acid groups (that are all carbon atoms bound to two oxygen atoms and another carbon atom).

With this set of reactive sites, we sampled a maximum number of two intermolecular bond formations and one bond dissociation. Reaction coordinates are constructed from the allowed combinations of formation and dissociation and the reactive sites. For each reactive site, CHEMOTON defines possible directions of attack as local minima of a repulsive potential sampled on a sphere around the reactive site. Each reactive site in our one-step exploration had only one possible direction of attack, except the carbon nucleus of the carboxylate group, which had three possible directions of attack. The bimolecular reactive complexes are formed by CHEMOTON by aligning the possible directions of attack. This leaves one more conformational degree of freedom, the rotation around the aligned directions of attack. In this ensemble, we select two rotamers that are rotated by 180 degrees relative to each other for reaction exploration. In the construction of reaction coordinates, every reactive site may form a bond with any other reactive site a priori, hence, also reactions that are contradictory to chemical intuition, such as the bond formation between the two hydrogen nuclei, were sampled in our short exploration. Further limitations could have been imposed on the reactive site combinations in our framework, but they were omitted in this work due to the computational efficiency of the underlying semiempirical electronic structure model. The combination of a maximum number of two bond formations and one bond dissociation with two rotamers each and three possible directions of attack for the carbon nucleus in the carboxylate group amounts to a total of 510 reaction trials. We have included the steering protocol with which we have carried out the exploration on Zenodo[11], making the exploration completely reproducible. We have also stored the resulting database in the same repository in order to make the subsequent QM/MM calculations based on individual elementary step database identifiers reproducible as the database identifiers include a time stamp and will not be identical in a re-run of the exploration.

QM core model exploration

Supplementary Table 1: Relative electronic energies for all elementary step IDs of all one-step esterifications in the core model discovered by CHEMOTON. The energies are calculated with PBE-D3/def2-SVP single point calculations on DFTB3 structures. R and P stands for the reactants and products respectively in an optimized complex (IRC endpoints), TS stands for transition state, E_A stands for activation and $\Delta_R E$ reaction energy respectively. All energies are relative to the lowest reactant energy. All energies are given in kcal mol^{-1}. Source data are provided as a Source Data file.

Elementary step	R	TS	P	E_A	$\Delta_R E$
63da313e8963666af14de41f	2.3	46.0	5.1	43.7	2.8
63da2f4c4fd2ee55fb0aa54f	4.8	46.8	3.2	42.0	-1.6
63da325f9a62416b752c276f	4.4	51.9	9.3	47.5	4.9
63da31d5943a3411230a6c6f	3.6	46.8	8.9	43.2	5.3
63da34639d046e7609024d3f	2.0	56.4	12.7	54.4	10.8
63da317afb6f81728b633c4f	2.0	51.5	17.9	49.4	15.9
63da3199adb2cd756704914f	6.7	49.7	4.4	43.0	-2.4
63da306c79b1a765242c276f	0.0	49.2	6.1	49.2	6.1
63da31e26fa128593d31241f	2.4	46.0	5.1	43.7	2.7
63da2f213d0fc3732d0524ff	0.0	49.2	6.2	49.2	6.1
63da3085f64605240274bacf	0.0	49.2	6.1	49.2	6.1
63da318e5aa2cd5a1c6d201f	4.8	46.8	3.4	42.0	-1.4
63da307627823463a329f31f	6.8	49.8	4.5	42.9	-2.4
63da2f901a285a7f641de51f	2.0	51.4	17.9	49.4	15.9
63da2f63bfa464166d6cefdf	0.0	49.2	6.2	49.2	6.2
63da304d056f994f093613df	2.0	56.4	12.7	54.4	10.7
63da30a0d748533173136cef	4.8	46.7	3.4	42.0	-1.3

Full QM model exploration

Supplementary Table 2: Relative electronic energies obtained from reaction trials with DFTB3 in the nanoscopic system. The identifiers refer to the elementary step discovered in the core model, from which the transplanted reactive complex served as the starting point for the reaction trial. The energies are calculated with PBE-D3/def2-SVP single point calculations on DFTB3 structures. R and P stands for the reactants and products respectively in an optimized reactive complex (IRC endpoints), TS stands for transition state, E_{A} stands for activation and $\Delta_{\mathrm{R}}E$ reaction energy respectively. All energies are relative to the lowest reactant energy. All energies are given in kcal / mol^{-1}. Source data are provided as a Source Data file.

Elementary step	R	TS	P	E_{A}	$\Delta_{\mathrm{R}}E$
63da31d5943a3411230a6c6f	33.4	71.5	25.2	38.1	-8.2
63da318e5aa2cd5a1c6d201f	0.0	45.4	-7.4	45.4	-7.4

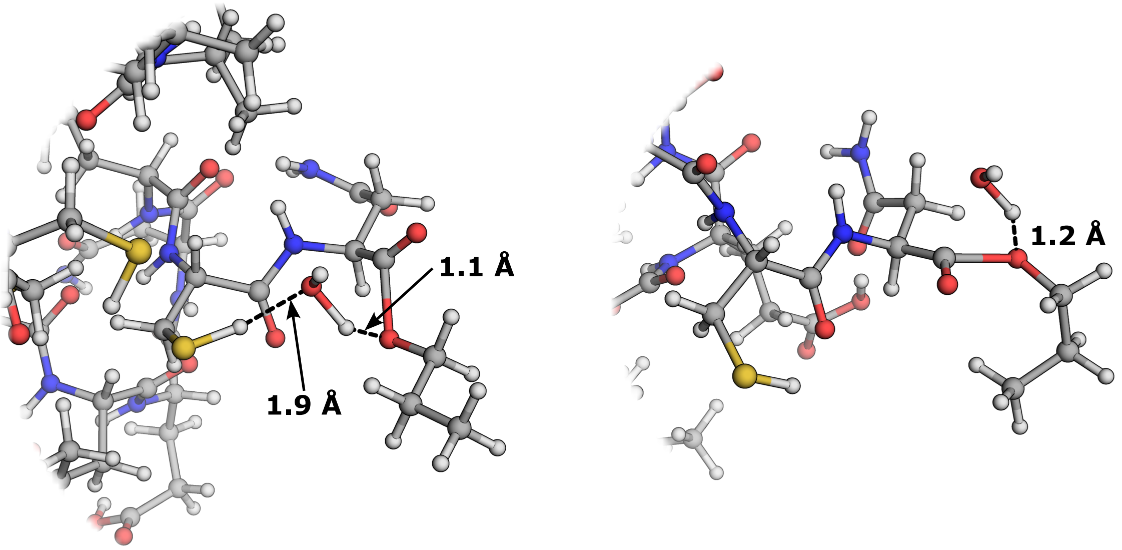

63da318e5aa2cd5a1c6d201f **63da31d5943a3411230a6c6f**

Supplementary Figure 4: Zoomed representation of structures of transition states of elementary steps “63da318e5aa2cd5a1c6d201f“ (left) and “63da31d5943a3411230a6c6f“ (right). Hydrogen bonds and correponding bond lengths are depicted as dashed black lines.

QM/MM exploration

Supplementary Table 3: Relative electronic energies obtained from reaction trials with DFTB3/SFAM in the nanoscopic system (SFAM: system-focused atomistic model). The identifiers refer to the elementary step discovered in the core model, from which the transplanted reactive complex served as the starting point for the reaction trial. The energies are calculated with PBE-D3/def2-SVP/SFAM single point calculations on DFTB3/SFAM structures. R and P stands for the reactants and products respectively in an optimized reactive complex (intrinsic reaction coordinate (IRC) endpoints), TS stands for transition state, E_{A} stands for activation and $\Delta_{\mathrm{R}}E$ reaction energy respectively. All energies are relative to the lowest reactant energy. All energies are given in kcal mol^{-1}. Source data are provided as a Source Data file.

Elementary step	R	TS	P	E_{A}	$\Delta_{\mathrm{R}}E$
63da313e8963666af14de41f	17.2	63.3	33.0	46.1	15.9
63da31d5943a3411230a6c6f	29.6	73.7	43.3	44.1	13.7
63da317afb6f81728b633c4f	0.8	47.3	16.9	46.5	16.1
63da3199adb2cd756704914f	12.4	59.6	21.4	47.2	9.0
63da31e26fa128593d31241f	26.0	70.5	39.9	44.5	13.8
63da3085f64605240274bacf	41.6	71.0	28.9	29.4	-12.7
63da318e5aa2cd5a1c6d201f	16.4	61.5	22.8	45.0	6.4
63da2f901a285a7f641de51f	0.0	46.1	15.2	46.1	15.2
63da2f63bfa464166d6cefdf	32.8	69.8	36.9	37.0	4.0

QM/MM IRC

Supplementary Table 4: Relative electronic energies obtained from IRC calculations starting from a transition state found by Chemoton in the core model and transplanted into a full structural model of the protein. The identifiers refer to the elementary step discovered in the core model, from which the transition state was taken. The energies are calculated with PBE-D3/def2-SVP/SFAM single point calculations on DFTB3/SFAM structures. R and P stands for the reactants and products respectively in an optimized reactive complex (IRC endpoints), TS stands for transition state, E_A stands for activation and $\Delta_\mathrm{R}E$ reaction energy respectively. All energies are relative to the lowest reactant energy of the DFTB3/SFAM exploration (see Supplementary Table 3). All energies are given in kcal mol^{-1}. Source data are provided as a Source Data file.

Elementary step	R	TS	P	E_A	$\Delta_\mathrm{R}E$
63da313e8963666af14de41f	17.6	66.9	30.6	49.3	13.0
63da2f4c4fd2ee55fb0aa54f	28.5	63.1	27.3	34.6	-1.2
63da325f9a62416b752c276f	5.6	63.8	11.2	58.2	5.6
63da31d5943a3411230a6c6f	38.4	73.1	39.5	34.7	1.1
63da34639d046e7609024d3f	45.1	79.9	39.9	34.9	-5.2
63da317afb6f81728b633c4f	-2.0	51.6	20.5	53.7	22.6
63da306c79b1a765242c276f	17.3	52.0	19.2	34.7	1.9
63da31e26fa128593d31241f	31.5	66.9	29.8	35.4	-1.7
63da2f213d0fc3732d0524ff	20.7	55.2	23.5	34.5	2.8
63da3085f64605240274bacf	21.3	55.0	23.1	33.7	1.9
63da318e5aa2cd5a1c6d201f	28.5	64.1	28.9	35.7	0.4
63da307627823463a329f31f	12.0	57.4	22.7	45.4	10.6
63da2f901a285a7f641de51f	1.9	53.9	23.4	52.1	21.5
63da2f63bfa464166d6cefdf	20.7	54.9	23.1	34.3	2.5
63da304d056f994f093613df	42.3	76.9	36.7	34.7	-5.5
63da30a0d748533173136cef	28.1	64.0	28.8	35.9	0.7

QM/MM stationary points

Supplementary Table 5: Relative electronic energies obtained with optimizations of the environment around the stationary points of the core model with DFTB3/SFAM. The identifiers refer to the elementary step discovered in the core model, from which the stationary points where transplanted into the protein which was subsequently optimized with the core model constrained. The energies are calculated with PBE-D3/def2-SVP/SFAM single point calculations on DFTB3/SFAM structures. R and P stands for the reactants and products respectively in an optimized reactive complex (IRC endpoints), TS stands for transition state, E_{A} stands for activation and $\Delta_{\mathrm{R}}E$ reaction energy respectively. All energies are relative to the lowest reactant energy of the DFTB3/SFAM exploration (see Supplementary Table 3). All energies are given in kcal mol^{-1}. Source data are provided as a Source Data file.

Elementary step	R	TS	P	E_{A}	$\Delta_{\mathrm{R}}E$
63da313e8963666af14de41f	14.3	58.0	24.5	43.7	10.2
63da2f4c4fd2ee55fb0aa54f	10.7	63.2	17.0	52.4	6.2
63da325f9a62416b752c276f	23.2	58.9	27.1	35.7	3.8
63da31d5943a3411230a6c6f	28.9	73.1	35.3	44.2	6.4
63da34639d046e7609024d3f	26.1	80.1	31.7	54.0	5.5
63da317afb6f81728b633c4f	6.4	71.2	38.4	64.8	32.0
63da3199adb2cd756704914f	19.5	64.7	25.9	45.2	6.4
63da306c79b1a765242c276f	20.0	55.8	14.2	35.8	-5.8
63da31e26fa128593d31241f	24.3	66.6	32.0	42.4	7.7
63da2f213d0fc3732d0524ff	24.7	69.2	15.3	44.5	-9.4
63da3085f64605240274bacf	22.8	56.0	12.2	33.1	-10.7
63da318e5aa2cd5a1c6d201f	12.5	63.9	9.8	51.4	-2.7
63da307627823463a329f31f	20.9	56.9	23.8	36.0	2.9
63da2f901a285a7f641de51f	10.8	61.0	39.0	50.2	28.2
63da2f63bfa464166d6cefdf	24.0	56.0	20.6	31.9	-3.4
63da304d056f994f093613df	16.8	76.5	29.9	59.6	13.1
63da30a0d748533173136cef	12.4	63.7	3.5	51.3	-8.9

Supplementary Table 6: Energy decomposition of the activation energy E_A and reaction energy $\Delta_\mathrm{R}E$ in the various structural models and exploration strategies for the elementary step with the database identifier "63da318e5aa2cd5a1c6d201f", which was the elementary step with the lowest reactant energy in the full QM exploration. The values given in parentheses correspond to the individual QM and MM contributions ($\Delta_\mathrm{R}E = \Delta_\mathrm{R}E^\mathrm{QM} + \Delta_\mathrm{R}E^\mathrm{MM}$, analogously for E_A). Root mean square deviations (RMSDs) between reactant and transition state structure (R−TS) and transition state and product (TS−P) are given in Ångstrom for the QM region, and for the MM region in parentheses and exclude hydrogen nuclei. The QM and MM region are fit by a quaternion fit separately from each other. In the case of the full QM exploration the RMSD of the MM region refers to the nuclei that were treated classically in the QM/MM models. Source data are provided as a Source Data file.

	$\Delta_\mathrm{R}E$ / kcal mol^{-1}	E_A / kcal mol^{-1}	RMSD/ Å	
			R−TS	TS−P
QM full model	−7.4 (−7.4 / −)	45.4 (45.4 / −)	0.6 (0.1)	0.8 (0.2)
QM core	−1.4 (−1.4 / −)	42.0 (42.0 / −)	1.1 (−)	1.2 (−)
QM/MM Exploration	6.4 (0.8 / 5.5)	45.0 (36.5 / 8.6)	0.7 (0.1)	0.5 (0.1)
QM/MM IRC	0.4 (2.8 / 3.3)	35.7 (30.7 / 5.0)	0.7 (0.1)	0.4 (0.0)
QM/MM stat. points	−2.7 (−1.3 / −1.3)	51.4 (42.0 / 9.4)	1.1 (1.3)	1.2 (1.5)

Supplementary Figure 5 illustrates the activation energies of all models in which the elementary step with the lowest reactant energy of the full QM exploration was used as input:

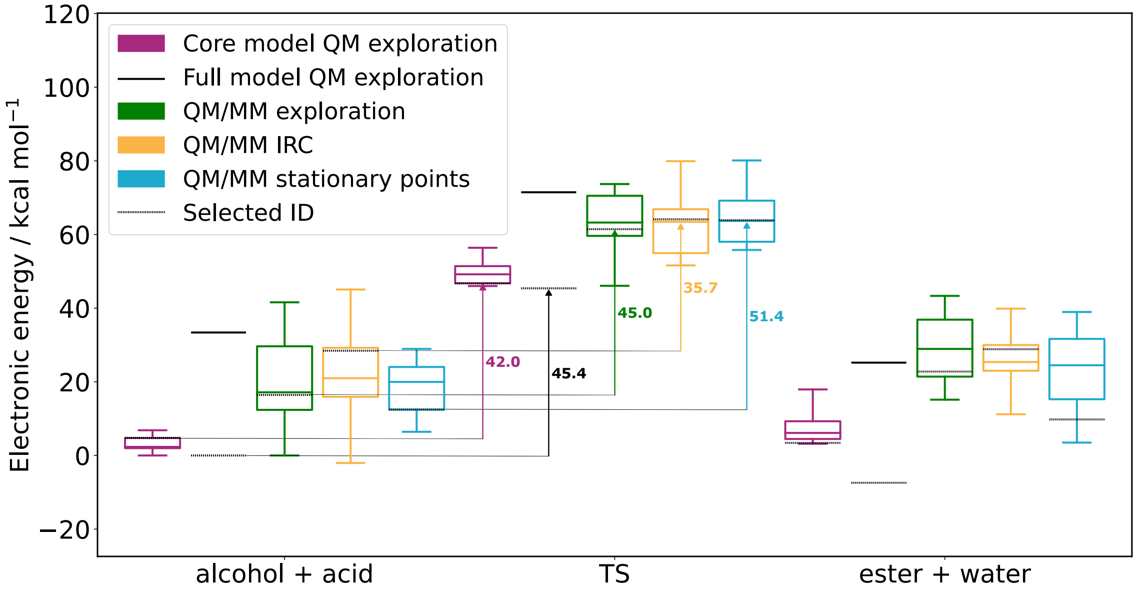

Supplementary Figure 5: Box plots for the spread of relative electronic energies of the stationary points of the reaction for the core model (n = 17), the 'QM/MM stationary points' (QM/MM: quantum mechanical/molecular mechanical) of the transplanted core model into the full structure with environment relaxation (n = 17), 'QM/MM IRC' obtained from an intrinsic reaction coordinate (IRC) scan starting from the transition state in the full QM/MM model (n = 16), 'QM/MM exploration' calculated in a single-ended reaction search in the full structural model starting from the reactive complex of the reactants (n = 9), and 'Full model QM exploration' calculated in a single-ended reaction search in the full structural model and a full QM description (n = 2). The energies were calculated with PBE-D3/def2-SVP/SFAM single-point calculations on DFTB3/SFAM structures (SFAM: system-focused atomistic model), and are given relative to the lowest reactant conformer found with each strategy. The whiskers of the boxes show the minimum and maximum values, the boxes depict the second and third quartile, and the line in the box denotes the median of the energy values. The dashed horizontal bar annotates the elementary step with the lowest reactant energy for the full QM model, and the respective elementary step in the QM/MM model for which the full-QM-model-step was used as an input. The activation energies for this elementary step in all approaches are annotated with vertical arrows. Source data are provided as a Source Data file.

Supplementary Table 7: Root mean square deviations (RMSDs) of the reactant, transition state, and product structures obtained with different approaches compared to those in the full QM model for the elementary step with the database identifier "63da318e5aa2cd5a1c6d201f". The values are given in Ångstrom for the QM region, and for the MM region in parentheses and exclude hydrogen nuclei. The QM and MM region are fit by a quaternion fit separately from each other. Source data are provided as a Source Data file.

Approach	Reactant	Transition state	Product
QM core	1.0 (−)	0.8 (−)	1.4
QM/MM exploration	0.5 (1.4)	0.5 (1.5)	1.2 (1.4)
QM/MM IRC	0.9 (1.2)	0.8 (1.2)	1.1 (1.4)
QM/MM stat. points	1.0 (0.9)	0.8 (1.7)	1.4 (1.2)

Supplementary Table 8: Energy decomposition of the activation energy E_{A} and reaction energy $\Delta_{\mathrm{R}}E$ in the various structural models and exploration strategies for the elementary step with the database identifier "63da2f63bfa464166d6cefdf", which was the elementary step with the lowest reactant energy in the core model. The values given in parentheses correspond to the individual QM and MM contributions ($\Delta_{\mathrm{R}}E = \Delta_{\mathrm{R}}E^{\mathrm{QM}}+\Delta_{\mathrm{R}}E^{\mathrm{MM}}$, analogously for E_{A}). Root mean square deviations (RMSDs) between reactant and transition state structure (R−TS) and transition state and product (TS−P) are given in Ångstrom for the QM region, and for the MM region in parentheses and exclude hydrogen nuclei. The QM and MM region are fit by a quaternion fit separately from each other. Source data are provided as a Source Data file.

	$\Delta_{\mathrm{R}}E$ / kcal mol^{-1}	E_{A} / kcal mol^{-1}	RMSD/ Å	
			R−TS	TS−P
QM core	6.2<br>(6.2 / −)	49.2<br>(49.2 / −)	1.2 (−)	1.4 (−)
QM/MM stat. points	−3.4<br>(6.2 / −9.6)	31.9<br>(49.2 / −17.3)	1.2 (1.4)	1.4 (0.7)
QM/MM IRC	2.5<br>(4.6 / −2.1)	34.3<br>(33.2 / 1.0)	0.5 (0.0)	0.4 (0.1)
QM/MM Exploration	4.0<br>(3.6 / 0.4)	37.0<br>(34.1 / 2.9)	0.6 (0.1)	0.5 (0.1)

4.2 Two-step reaction mechanism

We concurrently studied the following two-step esterification reaction mechanism with Scine Chemoton:

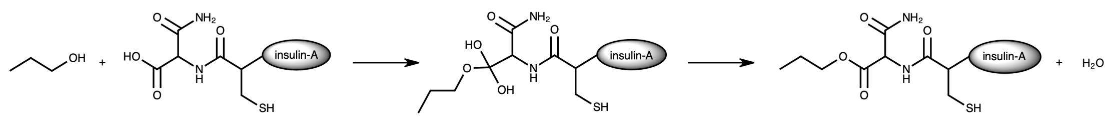

Supplementary Figure 6: Two-step reaction of 1-propanol with chain A of insulin (its backbone is represented by a gray circle) proceeding via a tetrahedral intermediate.

The tetrahedral intermediate is found in the same exploration step as the one-step esterification reaction with the reaction rules discussed in section 4.1. For the second step, we kept the same reaction rules and combined them with a `Lowest_Barrier_Selection` that allows the exploration of the products of the ten lowest reactions. We then proceeded with a `Rearrangement` network expansion step that samples only unimolecular reactions and again sampled a maximum of two bond formations and one bond dissociation. This yielded the expected product. In total, the steering protocol of the one-step mechanism reads: [`File_Input_Selection`, `Simple_Optimization`, `All_Compound_Selection`, `Association`,] and continues for the two-step mechanism: [`Lowest_Barrier_Selection`, `Rearrangement`]. For more details, on the nomenclature of different network expansion and selection steps and the carried out operations in these steps, we refer to ref. 12. Identical to the one-step mechanism, we store the steering protocol as well as the complete database on Zenodo[11]. In order to keep the two mechanisms separate, we have started the exploration of the two-step mechanism from scratch, which is why the database identifier of the one-step mechanism within the reaction network of the two-step mechanism is different to those discussed in section 4.1. All elementary steps that we found for the two reactions constituting the two-step mechanism are given below.

Supplementary Table 9: Relative electronic energies for all elementary step IDs of all two-step esterifications in the core model discovered by CHEMOTON. The energies are calculated with PBE-D3/def2-SVP single point calculations on DFTB3 structures. R and P stands for the reactants and products respectively in an optimized reactive complex (IRC endpoints), TS stands for transition state, E_A stands for activation and $\Delta_\mathrm{R}E$ reaction energy respectively. All energies are relative to the lowest reactant energy. All energies are given in kcal mol^{-1}. Source data are provided as a Source Data file.

First step					
Elementary step	R	TS	P	E_A	$\Delta_\mathrm{R}E$
65b80568cc9d215bd20b3fb8	0.0	48.2	17.5	48.2	17.5
65b8065281cb3658744a4028	1.2	48.0	16.5	46.8	15.3
65b809bb68b5d3709e1086a8	8.3	55.5	18.4	47.2	10.1
65b805a84f3b0201c1218d58	6.1	47.5	17.5	41.4	11.4
65b8055183b14f7638015678	6.1	47.5	17.6	41.4	11.5
65b80a011836e209cd02c3d8	0.0	41.8	13.7	41.8	13.7
65b8054a18ac245e3f7de1a8	5.8	48.1	12.3	42.4	6.6
65b8065c5dbfeb3a875d3f58	6.2	47.6	15.2	41.4	9.0
65b80597f939ef22f17e42b8	8.3	53.0	13.2	44.6	4.8
65b8052278c2d03136136548	1.3	45.2	15.6	43.9	14.2
65b8049629aeea11dd025578	6.2	47.6	15.1	41.4	8.9
65b80577a6385948ba14c7e8	13.3	48.0	12.9	34.8	-0.3
Second step					
Elementary step	R	TS	P	E_A	$\Delta_\mathrm{R}E$
65b8101b2342891c273c37c8	14.5	51.9	6.1	37.4	-8.4
65b80f3aa2e63613d346f4d8	12.8	48.2	5.6	35.4	-7.2
65b80f548c88aa777a0a4538	12.8	52.9	6.5	40.0	-6.3
65b8100236a246707b5950b8	12.6	60.8	23.5	48.2	10.8
65b80ff9844f7e3dd77a6068	13.0	60.8	23.5	47.9	10.5
65b80f55d9487a65535a1f18	12.8	52.9	6.5	40.0	-6.3
65b80f1cb88856031d51bd08	22.0	59.3	21.0	37.4	-0.9
65b80fcd0971713cba6d6fc8	16.2	60.9	23.5	44.7	7.3

The stationary states of the two elementary steps with the lowest-lying reactant energy each are displayed in Supplementary Figure 7.

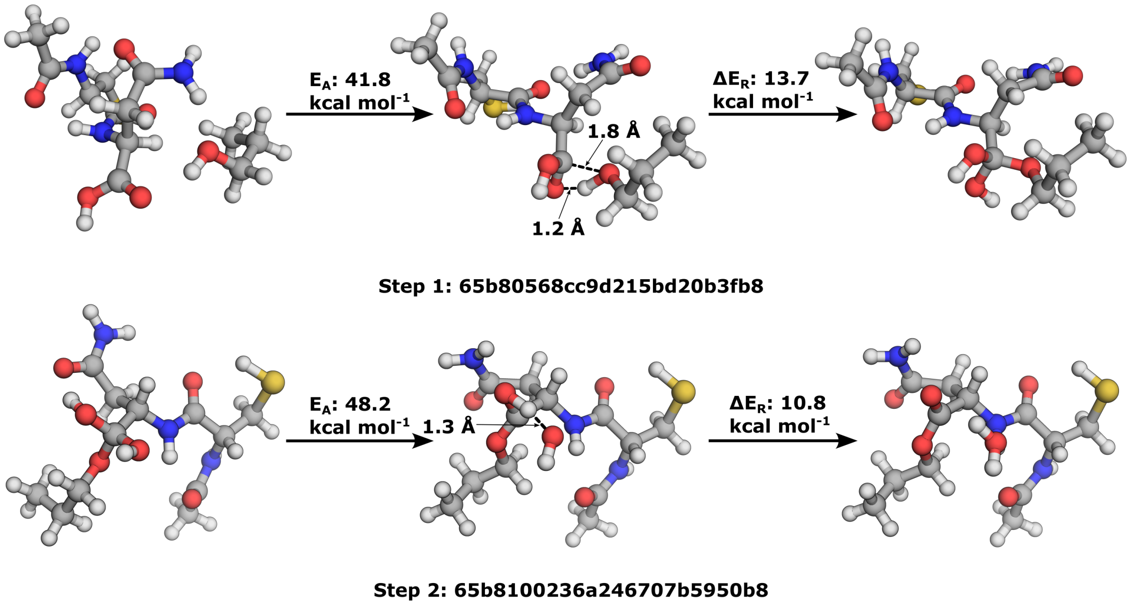

Supplementary Figure 7: Reactant (left), transition state (center) and product (right) molecular structures of the first elementary step “65b80568cc9d215bd20b3fb8“ (top) and “65b8100236a246707b5950b8“ (bottom) with the lowest-lying reactant energy each. Hydrogen bonds are depicted as dashed lines.

We have also applied our FUNNEL protocol with the QM/MM IRC calculations on the transplanted transition states from the core model as this offers the best balance between computational cost and accuracy. The multi-step nature of the two-step mechanism complicates the construction of an energy diagram as shown in Supplementary Figure 8, because the product side of the first step might not be the identical conformer as the reactant side of the second step. Therefore, we depict the energies of the intermediate for the two sides separately in Supplementary Figure 8.

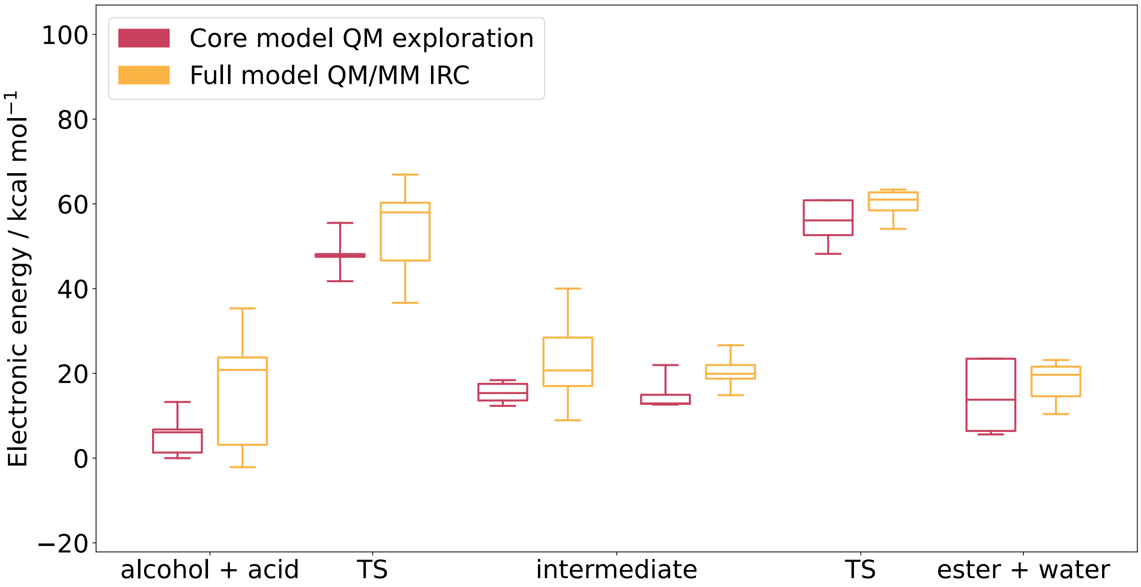

Supplementary Figure 8: The relative energies of the two-step esterification path in the core model and with our quantum mechanical/molecular mechanical (QM/MM) intrinsic reaction coordinate (IRC) approach, where the transition states of the core model are transplanted into the full model and then an IRC calculation is carried out. The energies of the intermediate are given twice, once for the product side of the first step and once for the reactant side of the second step as they originate from two separate IRC calculations. ’TS’ stands for ’transition state’.

Supplementary Table 10: Relative electronic energies obtained from IRC calculations starting from transition states of the two-step mechanism found by CHEMOTON in the core model and transplanted into a full structural model of the protein. The identifiers refer to the elementary step discovered in the core model, from which the transplanted transition state served as the starting point of the IRC calculation. The energies are calculated with PBE-D3/def2-SVP/SFAM single point calculations on DFTB3/SFAM structures. R and P stands for the reactants and products respectively in an optimized reactive complex (IRC endpoints), TS stands for transition state, E_A stands for activation and $\Delta_\mathrm{R}E$ reaction energy respectively. All energies are relative to the lowest reactant energy of the DFTB3/SFAM exploration (see Supplementary Table 3). All energies are given in kcal mol^{-1}. Source data are provided as a Source Data file.

First step					
Elementary step	R	TS	P	E_A	$\Delta_\mathrm{R}E$
65b80568cc9d215bd20b3fb8	16.6	50.8	20.3	34.2	3.8
65b8065281cb3658744a4028	25.3	60.3	29.3	35.0	4.0
65b809bb68b5d3709e1086a8	24.6	64.2	21.1	39.6	-3.6
65b805a84f3b0201c1218d58	20.6	59.8	28.4	39.2	7.8
65b8055183b14f7638015678	21.1	60.2	28.5	39.2	7.4
65b80a011836e209cd02c3d8	-0.5	36.6	8.9	37.2	9.5
65b8054a18ac245e3f7de1a8	35.3	66.9	40.0	31.6	4.7
65b8065c5dbfeb3a875d3f58	1.1	46.2	17.7	45.1	16.6
65b80597f939ef22f17e42b8	23.1	58.8	20.0	35.7	-3.2
65b8052278c2d03136136548	23.5	57.2	28.4	33.7	5.0
65b8049629aeea11dd025578	-2.1	43.0	14.8	45.1	16.9
65b80577a6385948ba14c7e8	3.8	46.8	9.5	42.9	5.6
Second step					
Elementary step	R	TS	P	E_A	$\Delta_\mathrm{R}E$
65b8101b2342891c273c37c8	26.6	62.5	18.4	35.9	-8.2
65b80f3aa2e63613d346f4d8	19.3	56.1	12.7	36.8	-6.6
65b80f548c88aa777a0a4538	21.8	63.3	22.2	41.5	0.4
65b8100236a246707b5950b8	14.9	54.1	10.4	39.2	-4.5
65b80ff9844f7e3dd77a6068	20.5	59.2	15.2	38.7	-5.3
65b80f55d9487a65535a1f18	22.5	63.4	21.4	40.9	-1.1
65b80f1cb88856031d51bd08	17.6	62.4	20.9	44.8	3.3
65b80fcd0971713cba6d6fc8	19.1	59.6	23.1	40.5	4.0

5 Software Details

The work presented here mainly relies on different modules of the SCINE software project[13] developed in our group. All SCINE software modules are available open-source. The main modules are:

1. SCINE SWOOSE[14], the SCINE module for QM/MM modeling.
2. SCINE HERON[15], the Graphical User Interface.
3. SCINE CHEMOTON[16] for autonomous reaction network exploration.
4. SCINE READUCT[17], a library that implements structure and reaction path optimization routines.

Furthermore, different functionalities are distributed across different SCINE modules, which are automatically installed if required:

1. SCINE SPARROW[18], a library that provides a wide range of semiempirical methods.
2. SCINE DATABASE[19], a wrapper for a MONGODB database.
3. SCINE XTB WRAPPER[20], a wrapper around xtb[21].
4. SCINE CORE[22], providing general (calculator) interfaces.
5. SCINE UTILITIES[23], a library incorporating multiple functionalities required across all SCINE modules.
6. SCINE MOLASSEMBLER[24], a library to facilitate conversions between Cartesian and graph representations of molecules.
7. SCINE PUFFIN[25], a calculation handler for SCINE CHEMOTON. Note that all SCINE modules can be installed when a Puffin instance is bootstraped.

6 Technical details

The activation energy E_{A} derived from the individual electronic energies of all non-interacting reactant molecules R_i and the transition state TS is given by

$$E_{\mathrm{A}} = E(\mathrm{TS}) - \sum_i E(R_i) \tag{2}$$

and the reaction energy $\Delta_{\mathrm{R}}E$ by

$$\Delta_{\mathrm{R}}E = \sum_i E(P_i) - \sum_i E(R_i). \tag{3}$$

We approximate the above definitions and evaluate reaction and activation energy as

$$E_{\mathrm{A}} = E(\mathrm{TS}) - E(\mathrm{Lhs}) \tag{4}$$

and

$$\Delta_{\mathrm{R}}E = E(\mathrm{Lhs}) - E(\mathrm{Rhs}), \tag{5}$$

thus neglecting the association energy of reactants and/or products from infinite distance.

6.1 Protein representation

The secondary structure of the protein environment in Figure 3 in the main text was inferred from the Cartesian Coordinates with OpenBabel[26] and rendered as a cartoon in PyMOL.[27].

6.2 Convergence criteria applied to explorations with Chemoton

Supplementary Table 11: Settings employed during structure optimizations and elementary step trial calculations. These settings correspond to the default settings in CHEMOTON version 3.1.0. For further explanations, we refer to the manuals and documentation of READUCT[17] and PUFFIN[25]. Source data are provided as a Source Data file.

Calculation Type	Setting	Value
Structure Optimization	`convergence_max_iterations`	1000
	`convergence_step_max_coefficient`	0.002
	`convergence_step_rms`	0.001
	`convergence_gradient_max_coefficient`	0.0002
	`convergence_gradient_rms`	0.0001
	`convergence_requirement`	3
	`convergence_delta_value`	1e-06
	`geoopt_coordinate_system`	`cartesianWithoutRotTrans`
	`bfgs_use_trust_radius`	`True`
	`bfgs_trust_radius`	0.4
Newton Trajectory Scan	`convergence_max_iterations`	600
	`nt_total_force_norm`	0.1
	`sd_factor`	1.0
	`nt_use_micro_cycles`	`True`
	`nt_fixed_number_of_micro_cycles`	`True`
	`nt_number_of_micro_cycles`	10
	`nt_filter_passes`	10
Transition State Optimization	`convergence_max_iterations`	1000
	`convergence_step_max_coefficient`	0.002
	`convergence_step_rms`	0.001
	`convergence_gradient_max_coefficient`	0.0002
	`convergence_gradient_rms`	0.0001
	`convergence_requirement`	3
	`convergence_delta_value`	1e-06
	`optimizer`	`Bofill`
	`geoopt_coordinate_system`	`cartesianWithoutRotTrans`
	`bofill_trust_radius`	0.2
IRC	`convergence_max_iteration`	100
	`sd_factor`	2.0
	`irc_initial_step_size`	0.3
	`stop_on_error`	`False`
	`convergence_step_max_coefficient`	0.002
	`convergence_step_rms`	0.001
	`convergence_gradient_max_coefficient`	0.0002
	`convergence_gradient_rms`	0.0001
	`convergence_delta_value`	1e-06
	`irc_coordinate_system`	`cartesianWithoutRotTrans`

IRC Endpoint Optimization	convergence_max_iterations	1000
	convergence_step_max_coefficient	0.002
	convergence_step_rms	0.001
	convergence_gradient_max_coefficient	0.0002
	convergence_gradient_rms	0.0001
	convergence_requirement	3
	convergence_delta_value	1e-06
	geoopt_coordinate_system	cartesianWithoutRotTrans
	bfgs_use_trust_radius	True
	bfgs_trust_radius	0.2
Product Optimization	convergence_max_iterations	1000
	convergence_step_max_coefficient	0.002
	convergence_step_rms	0.001
	convergence_gradient_max_coefficient	0.0002
	convergence_gradient_rms	0.0001
	convergence_requirement	3
	convergence_delta_value	1e-06
	geoopt_coordinate_system	cartesianWithoutRotTrans
	bfgs_use_trust_radius	True
	bfgs_trust_radius	0.4

References

[1] Head, J. D. Computation of vibrational frequencies for adsorbates on surfaces. *Int. J. Quantum Chem.* **65**, 827–838 (1997).

[2] Li, H. & Jensen, J. H. Partial Hessian vibrational analysis: the localization of the molecular vibrational energy and entropy. *Theor. Chem. Acc.* **107**, 211–219 (2002).

[3] Banerjee, A., Adams, N., Simons, J. & Shepard, R. Search for Stationary Points on Surfaces. *J. Phys. Chem.* **89**, 52–57 (1985).

[4] Bofill, J. M. Updated Hessian matrix and the restricted step method for locating transition structures. *J. Comput. Chem.* **15**, 1–11 (1994).

[5] Schlegel, H. B. Geometry Optimization. *WIREs Comput. Mol. Sci.* **1**, 790–809 (2011).

[6] Broyden, C. G. The Convergence of a Class of Double-rank Minimization Algorithms 1. General Considerations. *IMA J. Appl. Math.* **6**, 76–90 (1970).

[7] Fletcher, R. A new approach to variable metric algorithms. *Comput. J.* **13**, 317–322 (1970).

[8] Goldfarb, D. A family of variable-metric methods derived by variational means. *Math. Comput.* **24**, 23–26 (1970).

[9] Shanno, D. F. Conditioning of quasi-Newton methods for function minimization. *Math. Comput.* **24**, 647–656 (1970).

[10] Brunken, C. & Reiher, M. Self-Parametrizing System-Focused Atomistic Models. *J. Chem. Theory Comput.* **16**, 1646–1665 (2020).

[11] Csizi, K.-S., Steiner, M. & Reiher, M. Data set for the journal article "Quantum Magnifying Glass for Chemistry at the Nanoscale". (2024). URL https://doi.org/10.5281/zenodo.10697553.

[12] Steiner, M. & Reiher, M. Navigating chemical reaction space with a steering wheel (2023). URL https://arxiv.org/abs/2308.16499.

[13] Software for Chemical Interaction and Networks (SCINE). URL https://scine.ethz.ch/. Accessed in February 2024.

[14] Brunken, C., Csizi, K.-S. & Reiher, M. qcscine/swoose: Release 1.0.0 (2021). URL https://zenodo.org/record/5782877.

[15] Bensberg, M. *et al.* qcscine/Heron: Release 1.0.0 (2022). URL https://zenodo.org/record/7038388.

[16] Bensberg, M. *et al.* qcscine/Chemoton: Release 3.0.0 (2023). URL https://zenodo.org/record/7928104.

[17] Bensberg, M. *et al.* qcscine/Readuct: Release 5.0.0 (2023). URL https://zenodo.org/record/7928089.

[18] Bosia, F. *et al.* qcscine/Sparrow: Release 4.0.0 (2023). URL https://zenodo.org/record/7928079.

[19] Bensberg, M. *et al.* qcscine/Database: Release 1.2.0 (2023). URL https://zenodo.org/record/7928096.

[20] Grimmel, S. A., Sobez, J.-G., Steiner, M., Unsleber, J. P. & Reiher, M. qcscine/Xtb_wrapper: Release 2.0.0 (2023). URL https://zenodo.org/record/7928082.

[21] Bannwarth, C. *et al.* Extended Tight-Binding Quantum Chemistry Methods. *WIREs Comput. Mol. Sci.* **11**, e1493 (2021).

[22] Bosia, F. *et al.* qcscine/core: Release 4.1.0 (2023). URL https://zenodo.org/record/7569654.

[23] Baiardi, A. *et al.* qcscine/Utilities: Release 8.0.0 (2023). URL https://zenodo.org/record/7928050.

[24] Bensberg, M. *et al.* qcscine/Molassembler: Release 2.0.0 (2023). URL https://zenodo.org/record/7928074.

[25] Bensberg, M. *et al.* qcscine/Puffin: Release 1.2.0 (2023). URL https://zenodo.org/record/7928099.

[26] O'Boyle, N. M. *et al.* Open Babel: An Open Chemical Toolbox. *J. Cheminformatics* **3**, 33 (2011).

[27] Schrödinger, L. & DeLano, W. PyMOL 2.5.0 (2021). URL https://pymol.org/2/. Accessed February 2024.
